# Factors associated with complete immunization coverage in children aged 12–23 months in Ambo Woreda, Central Ethiopia

**DOI:** 10.1186/1471-2458-12-566

**Published:** 2012-07-28

**Authors:** Belachew Etana, Wakgari Deressa

**Affiliations:** 1 College of Health Science, Mekelle University, P. O. Box: 1871, Mekelle, Ethiopia; 2 Department of Epidemiology and Biostatistics, School of Public Health, Addis Ababa University, Addis Ababa, Ethiopia

**Keywords:** Complete immunization, Immunization, Ambo

## Abstract

**Background:**

Vaccination is a proven tool in preventing and eradicating communicable diseases, but a considerable proportion of childhood morbidity and mortality in Ethiopia is due to vaccine preventable diseases. Immunization coverage in many parts of the country remains low despite the efforts to improve the services. In 2005, only 20% of the children were fully vaccinated and about 1 million children were unvaccinated in 2007. The objective of this study was to assess complete immunization coverage and its associated factors among children aged 12–23 months in Ambo woreda.

**Methods:**

A cross-sectional community-based study was conducted in 8 rural and 2 urban kebeles during January- February, 2011. A modified WHO EPI cluster sampling method was used for sample selection. Data on 536 children aged 12–23 months from 536 representative households were collected using trained nurses. The data collectors assessed the vaccination status of the children based on vaccination cards or mother’s verbal reports using a pre-tested structured questionnaire through house-to-house visits. Bivariate and multivariate logistic regression analyses were used to assess factors associated with immunization coverage.

**Results:**

About 96% of the mothers heard about vaccination and vaccine preventable diseases and 79.5% knew the benefit of immunization. About 36% of children aged 12–23 months were fully vaccinated by card plus recall, but only 27.7% were fully vaccinated by card alone and 23.7% children were unvaccinated. Using multivariate logistic regression models, factors significantly associated with complete immunization were antenatal care follow-up (adjusted odds ratio(AOR = 2.4, 95% CI: 1.2- 4.9), being born in the health facility (AOR = 2.1, 95% CI: 1.3-3.4), mothers’ knowledge about the age at which vaccination begins (AOR = 2.9, 95% CI: 1.9-4.6) and knowledge about the age at which vaccination completes (AOR = 4.3, 95% CI: 2.3-8), whereas area of residence and mother’s socio-demographic characteristics were not significantly associated with full immunization among children.

**Conclusion:**

Complete immunization coverage among children aged 12–23 months remains low. Maternal health care utilization and knowledge of mothers about the age at which child begins and finishes vaccination are the main factors associated with complete immunization coverage. It is necessary that, local interventions should be strengthened to raising awareness of the community on the importance of immunization, antenatal care and institutional delivery.

## Background

Vaccination has been shown to be one of the most cost-effective health interventions worldwide, through which a number of serious childhood diseases have been successfully prevented or eradicated. The immunization campaign carried out from 1967 to 1977 by the World Health Organization (WHO) eradicated the natural occurrence of small pox [1]. Despite the efforts to improve vaccination services, approximately 27 million infants were not vaccinated against measles or tetanus in 2007 [1]. As a result, 2–3 million children are dying annually from easily preventable diseases, and many more fall ill [1]. Although estimated global routine measles vaccination coverage reached 82% in 2007, nearly 23.2 million children were unvaccinated, of which 15.3 million (65%) resides in eight countries mainly in Africa [2].

In Ethiopia, about 1million children were estimated to be unvaccinated [2] and about 16% under-five mortality has been attributed to vaccine preventable diseases [3]. Immunization is one of the national child survival strategies in the country to reach diphtheria-pertussis and tetanus (DPT3)/measles vaccination coverage 90% in 2010 [4]. It is also presented as the key strategy to achieving the Millennium Development Goals (MDGs) specially to reduce the child mortality [5] and proportion of children immunized against measles is one of the MDG indicators of health [6].

In Ethiopia, the Expanded Program on Immunization (EPI) routine schedule recommends that infants should be vaccinated with the following vaccines: one dose of Bacillus Calmettee-Guerin (BCG) vaccine at birth (or as soon as possible); three doses of diphtheria, pertussis and tetanus (DPT) with hepatitis B (HepB) and *Haemophilus influenza* type b (Hib) (DPT-HepB-Hib) at 6, 10 and 14 weeks of age; at least three doses of oral polio vaccine (OPV) - at birth, and at 6, 10 and 14 weeks of age; and one dose of measles vaccine at 9 months of age. Therefore, children are expected to be fully immunized by 12 months of age. Three doses of pentavalent replaced DPT in the Ethiopian EPI Program in 2005 [7,8].

Although the vaccination coverage through the EPI has been improved in Ethiopia, the incidence of measles has increased from 3.19/100,000 in 2009 to 7.35/100,000 in 2010 [9]. The Ethiopian Demographic and Health Survey (DHS) 2005 revealed that only 20% of children 12–23 months of age were fully vaccinated and 24% of children did not receive any vaccination [10]. Children were more likely to be vaccinated the first doses of vaccination than the third and the fourth doses in which 60% of children received BCG and from these only 35% of them received measles vaccine [10].

Study done in south and north Ethiopia identified showed that, mothers’ educational status, urban residence and perceived health care support are significantly associated with complete immunization coverage [11,12]. Study from in Mozambique, India and Bangladesh also showed utilization of maternal health care service like ANC, tetanus toxoid vaccination and institutional delivery is associated with complete immunization status of children [13-15].

In addition, low access to services, inadequate awareness of caregivers, missed opportunities, and high dropout rate are major factors contributing to low immunization coverage [16]. However, only few studies have assessed factors associated with complete immunization coverage. Therefore, the aim of this study was to assess factors affecting the immunization status among children 12–23 months of age in Ambo Woreda and to generate data that could be used for better planning and strengthening of immunization services.

## Methods

### Study area and population

The study was conducted in 8 rural and 2 urban kebeles (small administrative units) of Ambo Woreda (District) from January 15 to February 16, 2011. According to the 2007 census, the population of Ambo Woreda was 161,063 and children under the age of five years constituted about 18% of the total population. The Woreda had a total of 34 rural and 4 urban kebeles. About 70% of the population resides in rural areas. The Woreda is mainly inhabited by a population belonging to the Oromo ethnic group. Christianity constituted about 95% of the religious denominations. The woreda has 1 zonal hospital, 4 health centers, 35 health posts and 11 private clinics. According to the 2009/10 Woreda Health Office report, 76.6% of children were fully vaccinated.

### Study design and sample

The study used a community based cross-sectional study design and data were collected from children between 12–23 months old and their mothers. A sample of 541 children aged 12–23 months was calculated using a single proportion population formula with a 95% confidence level, 5% margin of error and 20% estimated immunization coverage rate in the study area [10]. A 10% non-response rate and a design effect of 2 were considered.

The total kebeles in the Woreda were initially stratified into rural and urban areas. Then, 8 rural and 2 urban kebeles were selected by lottery from the total kebeles in the woreda. The modified 2005 WHO EPI cluster sampling method [17] was employed to select study households. Each kebele was considered as one cluster. The lists and number of households could not be found for all selected rural kebeles. So, equal number of household with at least one child between 12–23 months of age was selected from each of rural kebeles. In urban kebeles the crowdedness of households was considered as a result 147 households were selected. In each kebele the first household was selected by randomly chosen from the central location of kebele, then counting the households along the directional line to the edge of kebele area and selecting randomly one. The subsequent households were selected, according to the inclusion criteria, based on the principle of the next nearest household. Households in the kebele were visited until the allocated sample size for the kebele was fulfilled.

### Data collection

Interviewers administered a pre-tested structured questionnaire initially developed in English and later translated into *Afan Oromo* was used for data collection. Some of the questions were adopted from demographic and health survey of Ethiopia [10]. The questionnaire included sections on: socio-demographic characteristics of mother and child, utilization of ANC, TT immunization and health institution delivery by mothers, child characteristics, and knowledge of mother on vaccination and vaccine preventable diseases, and immunization history of the child.

Respondents were interviewed in the households by trained nurses. The acceptability of the questions and logical structure were checked in the field during pre-testing. Data on immunization history was collected either from vaccination cards or mother’s verbal report. A household was eligible if a child between 12–23 months of age was available in the house. After a child aged between 12–23 months was identified from the household through house-to-house visits, mothers of the child were asked for the presence of child’s immunization card. In case where there two or more child aged between 12–23 months the youngest child was selected. For the child with immunization card, the information on the doses and types of vaccines was copied from the card. In the absence of vaccination card, mothers were asked for immunization history of the child. The number of doses the child took and its route of administration was the way of collecting immunization history of the child. Information on other variables was asked directly from the child’s mother. Mothers of children were also interviewed about their knowledge on vaccination and vaccine preventable disease.

### Data analysis

Data were entered into EPI Info version 3.5.1 and transferred to SPSS version 16 for analysis. Data entry, cleaning, processing, preliminary analysis and final write-up were done by the researchers. Frequencies and other descriptive statistics were used to describe the data and binary analysis was used to assess the association between independent and dependent variables. Odds ratios (ORs) and their 95% confidence intervals (CIs) were calculated. A p-value <0.05 was considered as statistically significant. Bivariate analysis was done to identify the crude association between dependent and independent variables. Then, all variables that showed statistical significance in the bivariate analysis were included in the multivariate logistic regression model to determine the factors associated with full immunization coverage among children aged 12–23 months old.

Full immunization status of the children (card plus mothers recall) was included in the logistic regression model as a dependent variable, while socio-demographic characteristics of the mother, child characteristics, mothers utilization of ANC, TT immunization and health institutional delivery, knowledge of mothers’ on age at which the child begins and finishes immunization, and number of sessions needed for a child were used as independent variables. Adjusted ORs with their 95% CIs were computed to determine the association.

### Ethical considerations

The study protocol was reviewed and approved by the Institutional Review Board of the College of Health Sciences at Addis Ababa University. Permission to undertake the study was obtained from every relevant authority in the Woreda. Verbal informed consent was obtained from the participants prior to participation in the study, and data collection was conducted confidentially.

### The following operational definitions were used

**Fully vaccinated**- A child between 12–23 months old who received one BCG, at least three doses of pentavalent, three doses of OPV and a measles vaccine.

**Partially vaccinated**- a child who missed at least one dose of the eight vaccines.

**Unvaccinated**- a child who does not receive any dose of the eight vaccines.

**Vaccinated**- a child who take at least one dose of the eight vaccines.

**Coverage by card only**: Coverage calculated with numerator based only on documented dose, excluding from the numerator those vaccinated by history.

**Coverage by card plus history**: Coverage calculated with numerator based on card and mother’s report.

## Results

### Characteristics of study participants

A total of 536 mothers of children aged between 12–23 months old were interviewed, with a response rate of 99%. Of the total 536 children, 51% were boys. The majority (14.7%) of the children were aged 12 months, with a mean age of 16.8 months. Above one third (36%) of them were born at health institutions and 73% of the children were from rural areas. All respondents were mothers of the target children. The mean age of the mothers was 27.6 (SD = 6) years, which ranging from 17 to 50 years (Table 1).

**Table 1:** Socio–demographic characteristics of mothers and child Ambo Woreda Ethiopia, January, 2011

**Variable**	**Frequency**	**Percent**
Educational status		
Illiterate	257	47.9
Primary	201	37.5
Secondary and above	78	14.6
Marital status		
Unmarried	29	5.4
Married	472	88.1
Divorced	18	3.4
Widowed	17	3.2
Occupation		
House wife	104	19.4
Gov. Employee	50	9.3
Merchant	65	12.1
Daily labourer	77	14.4
Farmer	240	44.8
Ethnicity		
Oromo	507	94.6
Amhara	29	5.4
Religion		
Orthodox	331	61.8
Protestant	160	29.9
Other	45	8.4
Mothers		
<20	59	11.0
21-30	351	65.5
31-40	114	21.3
40-50	12	2.2
Sex of child		
Male	274	51.1
Female	262	48.9
Child’s place of delivery		
Home	344	64.2
Health institution	192	35.8

Only 37.5% and 14.6% of the mothers attended primary education and secondary or higher, respectively. The majority of mothers (88.1%) were married and 19.4% were housewives in occupation. The principal activity of the study participants was agriculture (44.8%). The majority (95%) of the study population belonged to Oromo ethnic group and 61.8% were Orthodox Christians (Table 1).

### Immunization coverage by card plus recall

Out of the total surveyed children aged 12–23 months, vaccination card was only seen and confirmed for 224 (41.8%) children. Based on vaccination card plus recall, 409 (76.3%, 95% CI: 72.6-79.8) children received one or more of the eight recommended vaccines and 23.7% (95% CI: 20.2-27.4) having never been vaccinated. Only 35.6% (95% CI: 31.7-39.8) of children completed all the recommended vaccines and 218 (40.7%) received one or more vaccines, but did not complete all the recommended doses (Table 2).

**Table 2:** Immunization coverage by card and card plus mothers’ recall, Ambo Woreda Ethiopia, January, 2011 (n = 536)

	**Card plus recall**		**Card only**	
**Vaccine**	**Frequency**	Percent*	**Frequency**	Percent*
BCG	381	71.1	216	40.3
OPV1	400	74.6	216	40.3
OPV2	363	67.7	206	38.4
OPV3	291	54.3	198	36.9
pentavalent1	354	66.0	202	37.7
pentavalent2	317	59.1	198	36.9
pentavalent3	257	47.9	191	35.6
Measles	294	54.9	160	29.9
Unvaccinated	127	23.7	312	58.2
Fully vaccinated	191	35.6	149	27.8

*The percentages cannot added to hundred.

From the eight vaccines, OPV was the most frequently received vaccine. Particularly, OPV1 was taken by the majority of children (74.6%), followed by BCG (71.1%) and OPV2 (67.7%). OPV3 (54.3%) and pentavalent3 (47.9%) coverage were the least taken vaccines when compared with other vaccines (Table 2). Pentavalent dropout rate was 12.9% and BCG to measles vaccine dropout rate was 22.2%. The coverage showed a decrement from the first doses of vaccine to the last doses.

### Immunization coverage by card only

Coverage by card was calculated by taking children who had vaccine card as a numerator. From the total 536 surveyed children, 40.3% took OPV1 and BCG vaccines, followed by OPV2 (38.4%). Pentavalent1 was also taken by 36.9% of children, and 35.6% took pentavalent3 vaccine. Measles vaccine was taken by 29.9% of children and 27.8% were fully vaccinated by card only (Table 2).

### Knowledge of mothers about vaccination

Of the total respondents, about 96% heard about vaccination and vaccine preventable diseases. The majority of respondents (79.5%) knew that the objective of vaccinating children was to prevent disease. About 77% of the respondents cited less than three types of vaccine preventable diseases, while 23.3% mentioned four or more types of vaccine preventable disease (Table 3).

**Table 3:** Mother’s knowledge about vaccination in children by area of residence Ambo Woreda Ethiopia, January, 2011

**Mother’s knowledge**	**Area of residence**		**Total, n (%)**	**p-value**
	**Rural, n (%)**	**Urban, n (%)**		
Heard about vaccination and vaccine preventable diseases				
Yes	373 (69.6)	141 (26.3)	514(95.9)	0.987
No	16 (3)	6 (1.1)	22(4.1)	
Vaccination is used to prevent disease				
Yes	300 (56)	126 (23.5)	426(79.5)	0.028
No	89 (16.6)	21 (3.9)	110(20.5)	
Knew four or more types of vaccine preventable diseases				
Yes	112 (20.9)	13 (2.4)	125 (23.3)	<0.001
No	277 (51.7)	134 (25)	411 (76.7)	
Knew the age at which child immunization begins				
Yes	156 (29.1)	85 (15.9)	241 (45)	<0.001
No	233 (43.5)	62 (11.6)	295 (55)	
Knew the age at which child completes immunization				
Yes	246 (45.9)	116 (21.6)	362 (67.5)	0.001
No	143 (26.7)	31 (5.8)	174 (32.5)	
Knew the total number of sessions needed for child immunization				
Yes	81 (15.1)	56 (10.4)	137 (25.9)	<0.001
No	308 (57.5)	91 (17)	399 (74.4)	

P-value <0.05 is significant at 95% CI.

With regard to respondent’s knowledge about the age at which the child begins and finishes immunization, 45% of them knew the age at child immunization begins and 67.5% knew the age at the child finishes immunization. In addition to these, only one fourth (25.9%) of the respondents knew the session needed to complete the child immunization. The knowledge of the mother was significantly different by the area of residence for the objective of vaccination, knowledge of number of vaccine preventable diseases, sessions needed for complete immunization and age at child begins and completes immunization (Table 3).

### Factors associated with complete immunization coverage

Table 4 shows bivariate and multivariate logistic regression analyses of socio-demographic characteristics of mothers and child associated with complete immunization coverage (card plus recall) among children aged 12–23 months of age. Bivariate analysis showed that literate mother (2.8, 95% CI: 1.9-4.0), urban resident (2.99, 95% CI: 2.0, 4.4) and child’s place of delivery (4.4, 95% CI: 2.9, 6.4) are significantly associated with complete immunization status of children. Using multivariate logistic regressions, only child’s place of delivery showed statistically significant association (AOR: 2.1, 95% CI: 1.3-3.4).

**Table 4:** Association between socio-demographic characteristics of mothers and their children in relation to complete immunization coverage among children aged 12–23 months, Ambo Woreda Ethiopia, January, 2011

**Variable**	Child fully vaccinated, n (%)*		**Odds Ratio (95% CI)**	
	**Yes**	**No**	**Crude**	**Adjusted**
Age of the mother				
15-20	23 (4.3)	35 (6.5)	1	NI
21-29	99 (18.5)	192 (35.8)	0.8 (0.4-1.4)	
≥30	69 (12.9)	118 (22)	0.89 (0.5-1.63)	
Mother’s education				
Illiterate	61 (11.4)	196 (36.6)	1	1
Literate	130 (24.3)	149 (27.8)	2.8 (1.9-4)**	1.33 (0.8-2.1)
Mother’s marital status				
Married	172 (31.2)	300 (56.0)	1	NI
Others	19 (3.5)	45 (8.4)	0.7 (0.4-1.3)	
Mother’s occupation				
Housewife	44 (8.2)	60 (11.2)	1	NI
Others	147 (27.4)	285 (53.2)	0.7 (0.5-1.1)	
Sex of the child				
Male	93 (17.4)	181 (33.8)	1	NI
Female	98 (18.3)	164 (30.6)	1.2 (0.8-1.7)	
Child’s birth place				
Home	81 (15.1)	263 (49.1)	1	1
Health institution	110 (20.5)	82 (15.3)	4.4 (2.9, 6.4)**	2.1 (1.3-3.4)**
Child’s birth order				
First	37 (6.9)	65 (12.1)	1	NI
Second	42 (7.8)	68 (12.7)	1.08 (0.6, 1.9)	
Third or more	112 (20.9)	212 (39.9)	0.9 (0.6, 1.5)	
Area of residence				
Rural	111 (20.7)	278 (51.9)	1	1
Urban	80 (14.9)	67 (12.5)	2.99 (2.0, 4.4)**	1.5 (0.9-2.6)

*Numbers in parenthesis are percentages **Significant NI- Variable not included in the model.

Table 5 shows the association between mother’s knowledge of vaccination, ANC follow-up and TT immunization in relation to complete immunization coverage (card plus recall) among children aged 12–23 months of age. Bivariate analysis shows that children of mother who knew that vaccination is used to prevent disease (4.5, 95% CI: 2.5-7.9); total session needed to complete immunization (1.7, 95% CI: 1.1, 2.5) and knew age at the child begins (5.9, 95% CI: 3.9-8.7) and completes (10, 95% CI: 5.7, 17.7) immunization were more likely to be fully vaccinated. But numbers of vaccine preventable disease that mothers knew had no association with the complete immunization status of children. In addition, children of mothers who had followed ANC during their last pregnancy (6.8, 95% CI: 4.0-10) and received TT (4.9, 95% CI: 3.1- 7.7) vaccination were more likely to be fully vaccinated.

**Table 5:** Association between mother’s knowledge of vaccination, ANC follow-up and TT immunization in relation to complete immunization coverage among children aged 12–23 months, Ambo Woreda Ethiopia, January, 2011

**Characteristics**	Child fully vaccinated, n (%)*		**Odds Ratio (95% CI)**	
	**Yes**	**No**	**Crude**	**Adjusted**
Mother knew vaccination is used to prevent disease				
Yes	176 (32.8)	250 (46.6)	4.5 (2.5-7.9)**	1.4 (0.7-2.8)
No	15 (2.8)	95 (17.7)	1	1
Mother knew four or more types of vaccine preventable diseases				
Yes	45 (8.4)	80 (14.9)	1 (0.7-1.5)	NI***
No	146 (27.2)	265 (49.4)	1	
Mother knew the age at which child immunization begins				
Yes	137 (25.6)	104 (19.4)	5.9 (3.9, 8.7)**	2.9 (1.9-4.6)**
No	54 (10.1)	241 (45)	1	1
Mother knew the age at which child immunization completes				
Yes	176 (32.8)	186 (34.7)	10 (5.7, 17.7)**	4.3 (2.3-8)**
No	15 (2.8)	159 (29.7)	1	1
Mother knew the total number of sessions needed for child immunization				
Yes	61 (11.4)	76 (14.2)	1.7 (1.1, 2.5)**	0.7 (0.4-1.2)
No	130 (24.3)	269 (50.2)	1	1
Mother had ANC follow-up				
Yes	165 (30.8)	172 (32.1)	6.8 (4.0, 10)**	2.4 (1.2-4.9)**
No	26 (4.9)	173 (32.3)	1	1
Mother received three or more doses of TT				
Yes	163 (30.4)	187 (34.9)	4.9 (3.1, 7.7)**	1.2 (0.6-2.4)
No	28 (5.2)	158 (29.5)	1	1

*Numbers in parenthesis are percentages, **Significant, *** NI- Variable not included in the model.

Multivariate analysis also shows that mother knowledge on age at the children vaccination begin (AOR, 2.9, 95% CI: 1.9-4.6) and completes immunization (AOR, 4.3, 95% CI: 2.3-8), ANC follow up (2.4, 95% CI: 1.2-4.9) were significantly associated with complete immunization status of children.

## Discussion

This study assessed the complete immunization coverage and factors associated with it among children aged between 12–23 months old in Ambo Woreda of Oromia Regional State found in Ethiopia. Based on immunization card and recall, 35.4% children were fully vaccinated, and 23.7% were unvaccinated. The pentavalent3 coverage was 47.9% and 54.9% took measles. The OPV vaccine coverage was slightly higher than the coverage of the pentavalent vaccine. The measles coverage was higher than the pentavalent3 coverage. But it is expected that the pentavalent3 coverage should be higher because of dropout and the long time gap between the two vaccines, in which the mother may not return back the measles vaccine. The higher coverage of measles and OPV vaccination was assumed to be due to the frequent national campaign that focused on the two vaccines.

Compared the immunization coverage of Ambo Woreda with the EDHS 2005, the proportion of children fully vaccinated in the present study was higher by 15%, but it was similar by proportions of unvaccinated children [10]. Beside this, the current findings is higher than the 2006 immunization coverage survey in the country which estimated fully vaccinated by card is only at 20% [18]. But it is lower than the immunization coverage reported in the 2008 health and health related indicators [19] and 2010 woreda health office report. This difference is may be due to the over reporting of health and health related indicators data from some areas.

From the total interviewed households, 224 (41.8%) mothers showed the vaccination card of their children. From the card most children took BCG and OPV1 vaccines, but only 27.8% of finished the immunization. The coverage by card only was also less than that of health and health related indicators. The proportion of fully vaccinated children in this study was about 8% higher than the EDHS 2005 and 2006 EPI survey coverage [10,18,19]. This difference is because of the result from such country level study includes area of low immunization coverage.

Apart from this, in this study mothers’ knowledge on vaccination and vaccine preventable disease was also assessed. About 96% mothers heard about child immunization and vaccine preventable disease, but only 79.5% of them mentioned that vaccination is used to prevent disease. About 20% of respondents knew four and above disease which are preventable vaccines. Regarding knowledge of mothers about the age at which the child begins and finishes the immunization, less than half of the respondents knew the correct age at the child begin immunization; and 67.5% of them knew the correct age at which the child should finish vaccination. In addition, only a fourth knew the number of sessions needed to complete immunization. The knowledge of mother is significantly different for respondents from urban and rural areas. This finding is consistence with the study done in Nigeria in which, more than half of mothers knew the purpose of immunization, but it is not similar with this study for the knowledge of the schedule of immunization [20]. The explanation may be related to the educational status of the mothers in which most of them are illiterate this study.

In this study age of mother, educational status, marital status, occupation and place of residence of the mothers did not show significant association with the completion of immunization among children aged between 12–23 months. This finding is similar with the case control study done in Wonago Woreda, Southern Ethiopia [12]. However, previous studies done in Sudan and other part of Ethiopia indicate that these factors have a significant association with completion of child immunization [11,21-23].

However, in this study children delivered at health facilities were more likely to be fully vaccinated (OR = 2.6) than children delivered at home. This finding was similar with the study done in Mozambique in which children delivered at home were less likely to complete immunization [13]. The explanation related to this may be that, mothers who gave birth at health institution are closer to the health service and most of the time the first dose of vaccination is given just after birth in health institution.

Besides its relation with institution delivery, complete immunization coverage of children showed statistically significant association with mothers’ utilization of antenatal care (ANC) follow up. Children of mother who had ANC were 2.1 times more likely to complete vaccination than those with no follow up. This is consistent with the study done in India and Bangladesh in which ANC follow up is related with complete immunization coverage [14,15].

In Ethiopia it was indicated that, lack of awareness about immunization contribute to low immunization coverage in Ethiopia [16]. The findings of this study also showed that lack of knowledge about vaccination and vaccine preventable disease, and age at the child begins and finishes the immunization, and is related with completion of immunization among children aged 12–23 months. Children of mothers those knew the correct age at which the child begins and finishes immunization are more likely to complete immunization than those who did not knew. These finding is consistent with the study findings from Tigray and Wonago in Ethiopia [12,18]. However, knowledge of objective of vaccination had no significant association with complete immunization. This indicates that knowledge of mothers about the schedule of immunization is more important in completion of the recommended immunization for the child than knowledge of the purpose of vaccination.

This study assessed full immunization coverage among children aged 12–23 months with regard to the recent immunization program performance. However it has certain limitations.

Report by the mother may under/overestimate the immunization coverage. Because mother may forgot the total doses of vaccine that the child took. Beside, this study did not consider the validity of the doses of vaccines child took. Also, sampling procedure was susceptible to selection bias and the study did not include qualitative method to answer the why questions. Moreover, problems from the health facility perspectives were not addressed by this study. Despite the above limitations, our findings are important to understand factors associated with immunization completion among children**.**

## Conclusion

Full immunization coverage among children aged 12–23 months remains very low in the woreda. Beside this, there is low awareness among mothers about age at child immunization begins and completes immunization as well as total sessions needed to complete immunization. Antenatal care follow up by mothers, health care institution delivery and mothers’ knowledge about age at child immunization begun and completed were found to be independent predictors for full child immunization status of children in the woreda.

Therefore, local action should be taken to raise awareness by designing proper health education targeting the mother on benefit, correct age child immunization begin and complete as well as session needed to complete immunization. In addition, the concerned body should work to increase utilization of antenatal care follow up and health care institution delivery which interns increase immunization coverage.

## Competing interest

We declare that we have no competing interest.

## Authors’ contribution

We have been contributed to this work equally. Both authors read and approved the final manuscript. All authors read and approved the final manuscript.

## Pre-publication history

The pre-publication history for this paper can be accessed here:

http://www.biomedcentral.com/1471-2458/12/566/prepub
